# Plant-based protein consumption patterns among Saudi Generation Z: a cross-sectional study of dietary frequencies, health outcomes, and sustainable eating behaviors

**DOI:** 10.3389/fpubh.2026.1739641

**Published:** 2026-01-20

**Authors:** Hala Hazam Al-Otaibi, Sawsan Al-Hashim

**Affiliations:** 1 Department of Food and Nutrition Science, College of Agricultural and Food Science, King Faisal University, Al-Ahsa, Saudi Arabia; 2 Department of Clinical Nutrition, College of Applied Medical Sciences, King Faisal University, Al-Ahsa, Saudi Arabia

**Keywords:** Arab region, dietary transition, environmental awareness, Generation Z, gulf countries, plant-based protein, Saudi Arabia, sustainable healthy diets

## Abstract

**Background:**

The global shift toward sustainable food systems has highlighted plant-based proteins as essential for environmental and health benefits. However, research on plant-based protein consumption among young adults in the Gulf region, particularly in Saudi Arabia, remains limited despite the country’s youthful demographic profile and increasing environmental awareness.

**Objective:**

This cross-sectional study explored plant-based protein consumption patterns frequencies among Saudi Generation Z (Gen Z) aged 18–25, examining associations with health outcomes, sustainable healthy eating behaviors (SHEBs), and readiness to adopt sustainable healthy diets.

**Methods:**

A non-representative convenience sample of 398 Saudi adults was recruited through snowball sampling. Data was collected via an online survey incorporating validated instruments for dietary consumption frequency, SHEB assessment, and stages-of-change measurement. Participants were categorized into three groups based on weekly consumption frequency of plant-based protein sources: low frequency (0–7 portions/week), moderate frequency (8–21 portions/week), and high frequency (≥22 portions/week). Statistical analyses included analysis of variance, chi-square tests, and binary logistic regression.

**Results:**

The median weekly consumption frequency of plant-based protein sources was 14 portions/week (IQR: 7–24), compared to 28 portions/week (IQR: 21–35) for animal-based protein sources. Only 21.4% of participants achieved high consumption frequency (≥22 portions/week). Higher plant-based protein consumption frequency was associated with lower mean body mass index (BMI) (23.19 ± 6.58 vs. 24.57 ± 7.02 kg/m^2^ among low-frequency consumers, *p* = 0.031), greater engagement in SHEBs (mean score: 2.17 ± 0.43 vs. 1.91 ± 0.38, *p* < 0.001), and more advanced stages of change toward adopting plant-based diets (*p* < 0.001). Health motivations predominated across all groups (>35%), while environmental concerns remained minimal (4.9–7.1%). The majority of participants (83.2%) were in the precontemplation or contemplation stages regarding plant-based diet adoption.

**Conclusion:**

Plant-based protein consumption among this Saudi Gen Z convenience sample remains markedly lower than animal-based protein intake, indicating limited integration of legumes, nuts, and plant-based alternatives into routine diets. Higher consumption frequency was observed alongside lower BMI and greater engagement in sustainable eating behaviors, suggesting associations that warrant further study. However, the cross-sectional design limits causal interpretation, and consumption frequency reflects behavioral patterns rather than validated nutritional intake or adequacy. Promoting health benefits while respecting traditional food practices may support healthier dietary shifts among young Arabs.

## 1 Introduction

The global food system faces unprecedented challenges in the 21st century, with mounting concerns about environmental degradation, climate change, and food security necessitating urgent dietary transformations (1). Current food production systems are responsible for approximately 26% of global greenhouse gas emissions, consume about 70% of freshwater resources, and occupy nearly 50% of habitable land, contributing to biodiversity loss and ecosystem degradation (2). These environmental pressures, combined with population growth and the rising prevalence of diet-related diseases, underscore the urgent need for sustainable dietary solutions.

Plant-based protein consumption has emerged as a promising pathway toward sustainable food systems, offering substantial environmental benefits while maintaining nutritional adequacy when properly planned (3). Compared with animal proteins, plant-based proteins require significantly less land, water, and energy resources, while producing lower greenhouse gas emissions and exerting less environmental impact (2). These advantages align closely with several Sustainable Development Goals (SDGs), particularly SDG 2 (Zero Hunger), SDG 3 (Good Health and Well-being), SDG 12 (Responsible Consumption and Production), SDG 13 (Climate Action), and SDG 15 (Life on Land). From a nutritional standpoint, plant proteins from legumes, nuts, and seeds can provide all essential amino acids when consumed as part of a varied diet. They are also associated with reduced risks of cardiovascular disease, type 2 diabetes, and certain cancers (4).

Recognizing these dual benefits, international health and nutrition organizations increasingly emphasize plant-based proteins as core components of sustainable healthy diets (SHDs). The Food and Agriculture Organization (FAO) and World Health Organization (WHO) define SHDs as “dietary patterns that promote all dimensions of individuals’ health and well-being; have low environmental pressure and impact; and are accessible, affordable, safe, and equitable, and culturally acceptable” (5). The EAT-Lancet Commission’s planetary health diet specifically recommends that global diets consist primarily of plant foods, including vegetables, fruits, whole grains, legumes, nuts, and unsaturated oils, supplemented with modest amounts of animal proteins. Adhering to this diet could prevent an estimated 11 million deaths annually while maintaining planetary boundaries for food production (1).

Evidence further indicates that health impacts vary by protein source. Higher consumption of animal-based protein, particularly from processed and red meats, is associated with an increased risk of being overweight and obese (6). In contrast, plant-based protein intake is positively linked to beneficial outcomes such as reduced weight (7).

Generation Z (Gen Z), individuals born between 1997 and 2012, represents a unique demographic cohort with characteristics directly relevant to sustainable food adoption (8). Research shows that this generation demonstrates heightened environmental consciousness, greater health awareness, and stronger motivation toward sustainable consumption patterns compared with earlier generations (9). Culliford and Bradbury (10) found that individuals under 35 were significantly more likely than older adults to reduce red meat consumption and to prioritize plant proteins as environmentally beneficial. Therefore, Gen Z represents a critical demographic for sustainable dietary transitions, as food preferences and behaviors established during young adulthood often persist throughout life, shaping long-term sustainability outcomes and serving as catalysts for broader societal dietary change.

Understanding dietary behavior change and Gen Z engagement with SHDs requires recognizing that adoption is a gradual process. Individuals typically move through stages of readiness before fully embracing new eating patterns. The Transtheoretical Model (TTM), or Stages of Change, provides a structured framework for this progression, spanning from precontemplation to maintenance. Several studies have applied this model to assess sustainability-related actions among Gen Z, including reductions in red and processed meat consumption and increased intake of plant-based diets (11, 12).

Saudi Arabia presents a particularly compelling context for examining plant-based protein adoption among Gen Z. The country has a notably young demographic profile, with more than 60% of the population under 30 years of age, creating a substantial Gen Z cohort (13). Islamic principles encourage dietary diversity and the consumption of wholesome foods, offering a religious framework compatible with culturally adapted plant-forward diets. However, Saudi culture traditionally emphasizes meat-based hospitality, where serving meat dishes to guests is a deeply ingrained practice, and omnivorous diets remain the norm. This cultural dynamic creates a unique opportunity to study how global sustainability trends intersect with traditional practices among environmentally conscious young Saudis.

In Saudi Arabia and the wider Gulf region, where dietary patterns remain heavily centered on animal-based proteins, direct transitions to fully plant-based diets encounter resistance. Evidence suggests, however, that gradually increasing plant-based protein intake can effectively reduce reliance on animal-based proteins. A flexitarian approach, emphasizing incremental increases in plant-based protein, appears to be the most effective strategy for promoting dietary change in this cultural context (1).

Globally, the prevalence of plant-based food consumption, including vegetarianism, varies widely, ranging from about 1% in countries such as Italy, China, and New Zealand (14–16), to more than 30% in South Asia and up to 40% in India (17, 18). In contrast, data from Middle Eastern and Gulf countries, including Saudi Arabia, remain limited, representing a significant gap in our understanding of plant-based dietary patterns in this region. Existing research suggests that while health, ethical, and environmental concerns motivate some Saudis to adopt plant-forward diets, the majority continue to follow traditional dietary patterns. Among those adopting vegetarian practices, women constitute the majority, reflecting broader awareness of the relationship between diet and health (19–21). Research from other Gulf and Middle Eastern countries is similarly scarce, with just a handful of studies conducted in settings that share comparable sociocultural and religious contexts, including Kuwait (22), the United Arab Emirates (23, 24), and Jordan (25).

This cultural context, combined with the lack of regional data, creates a compelling tension between traditional food practices and emerging global sustainability trends, particularly among environmentally conscious young Saudis. Despite global interest in plant-based diets and the documented environmental awareness of Gen Z, research on plant-based protein consumption patterns among Saudi Gen Z remains scarce. Few studies have directly assessed these demographics’ relative plant-protein consumption patterns, motivations for dietary choices, anthropometric measurements, or readiness to adopt SHDs within the Saudi cultural context. Understanding these factors is essential to identifying both barriers and facilitators that could inform culturally appropriate strategies for promoting SHD transitions and increasing plant-based protein consumption.

Therefore, this study aims to provide preliminary insights from a convenience sample of Saudi Gen Z participants into: (1) consumption frequency patterns of plant-based protein sources among a sample of Saudi Gen Z participants; (2) health status, measured using body mass index (BMI), and motivations influencing their plant-protein consumption frequency; (3) readiness to adopt SHD practices; and (4) associations between plant-based protein consumption frequency levels and SHD adoption in this population. By addressing these objectives, this study explores how cultural, social, and individual factors may influence sustainable eating behaviors among young adults in the Gulf and Arab regions, ultimately informing evidence-based interventions that promote healthier and more sustainable dietary patterns in Saudi Arabia.

## 2 Methods

### 2.1 Sampling procedure

A non-representative convenience sample of Saudi adults aged 18–25 years was recruited using a non-probability snowball sampling approach. This method was chosen for feasibility and accessibility; however, we acknowledge that this sampling strategy produces substantial selection bias and severely limits the generalizability of findings beyond our specific sample. The survey was initially distributed through university student networks and community groups in Saudi Arabia via WhatsApp, with participants encouraged to share the survey link within their own social networks. This recruitment approach resulted in a sample heavily skewed toward females (74.1%) and individuals with university-level education or higher (68.8%), demographic characteristics that do not reflect the broader Saudi Gen Z population.

Data collection was conducted via a Google Forms survey distributed through WhatsApp between November 2023 and March 2024 (*N* = 398). Eligibility criteria were limited to Saudi citizens who provided informed consent after reviewing the study protocol. To broaden the participant pool, respondents were encouraged to share the survey link within their networks.

The required sample size was calculated for a cross-sectional study comparing three groups based on frequency of plant-based protein consumption (low, moderate, and high). Given the continuous nature of the primary outcome (daily consumption frequency of plant-based protein intake), a one-way analysis of variance (ANOVA) was assumed. Calculations were performed using a significance level (*α* = 0.05), statistical power (1 – *β* = 0.80), and a medium effect size (Cohen’s *f* = 0.25), parameters commonly applied in behavioral nutrition research. Under these assumptions, a minimum of 159 participants (approximately 53 per group) was required. To account for potential nonresponse or incomplete data, the target sample size was increased by 15%, resulting in a final estimate of approximately 180 participants (60 per group) (26). The Scientific Research Ethics Committee of King Faisal University approved this study (KFU-REC-2023-JAN-ETHICS483) and was conducted in accordance with the ethical principles of the Declaration of Helsinki.

### 2.2 Study questionnaire

The questionnaire consisted of six sections adapted from validated instruments and underwent forward–backward translation by bilingual experts to ensure cultural relevance. A pilot test with 20 Saudi adults from Gen Z confirmed clarity, feasibility, and appropriateness, requiring only minor wording adjustments. Average completion time was 10–15 min. Pilot participants were excluded from the final analysis.

#### 2.2.1 Demographic data and anthropometric measurements

This section collected information on gender, age, educational level, monthly family income, and marital status. Participants self-reported their height and weight, which were used to calculate BMI and classify it according to WHO guidelines (27).

#### 2.2.2 Daily consumption of plant- and animal-based protein

Plant-based and animal-based protein consumption frequency patterns were assessed using a culturally adapted food frequency questionnaire (FFQ) based on the instrument developed by Hu et al. (28). The adapted FFQ focused on consumption frequency of specific food categories rather than validated protein quantification. The FFQ included the following plant-based protein sources commonly consumed in Saudi Arabia: cooked legumes (lentils, chickpeas, fava beans, kidney beans), raw nuts and seeds (almonds, walnuts, peanuts), plant-based meat substitutes (veggie burgers, plant-based nuggets), and plant-based milk (soy milk, almond milk). Animal protein sources included: red meat (beef, lamb), poultry (chicken, turkey), fish and seafood, eggs, and dairy products (milk, yogurt, cheese). Frequency options ranged from “four or more per day” (28 portions/week) to “rarely or never” (0 portions/week). An item-level upper cap of 35 portions/week (5 portions/day) was applied to each FFQ food category to limit extreme overreporting, following the approach of Hagmann et al. (29), and does not represent a nutritional intake threshold (see Supplementary Appendix A for full coding procedures).

Total weekly consumption frequency was calculated separately by summing the portions of animal and plant-based sources across all food categories. Participants were categorized into three plant-based protein consumption frequency groups based on tertile distributions: Low frequency: 0–7 total portions/week of plant-based protein sources; Moderate frequency: 8–21 total portions/week of plant-based protein sources; High frequency: ≥22 total portions/week of plant-based protein sources. These categories represent relative consumption frequency patterns within our sample rather than validated nutritional intake levels. The ratio of animal to plant-based protein consumption frequency was calculated as the ratio of total portions per week. Ratios are presented as descriptive indicators of relative consumption patterns within our sample and should not be interpreted as validated nutritional comparisons.

This FFQ measures consumption frequency of selected food categories not validated protein intake in grams or nutritional adequacy. Grains, vegetables, and mixed dishes were not systematically captured, which means total plant-based protein consumption frequency is likely underestimated. All participants included in the final analysis provided complete FFQ responses for both animal and plant-based protein items. Complete details of the FFQ instrument are provided in Supplementary Appendix A.

#### 2.2.3 Stage of change

Participants reported their adherence to a plant-based diet by selecting one of six statements adapted from TTM (Stages of Change) (30):

- Precontemplation (PC): “I am not interested in following a plant-based diet at present or in the future.”
- Contemplation (C): “I am currently thinking about following a plant-based diet and may start within the next 6 months.”
- Preparation (P): “I have decided to follow a plant-based diet in the near future.”
- Action (A): “I currently follow a plant-based diet.”
- Maintenance (M): “I have been following a plant-based diet for more than 6 months.”
- Relapse (R): “I used to follow a plant-based diet, but I have now stopped.”

For clarity, participants were provided with a brief definition stating that a “plant-based diet” refers to a dietary pattern that emphasizes plant-origin foods such as legumes, nuts, seeds, plant-based alternatives, and plant-based milks, without necessarily requiring the complete exclusion of animal products.

The distribution of responses across the six original stages was as follows: PC = 268 (67.3%), C = 63 (15.8%), *p* = 38 (9.5%), A = 9 (2.3%), M = 6 (1.5%), and R = 14 (3.5%). To address small cell sizes and align with established behavior change research, responses were combined into three theoretically meaningful groups for statistical analysis: early stages (PC + C), transition stages (P + R), and established adoption (A + M). This grouping reflects the non-linear nature of behavior change and accounts for individuals who may cycle between preparation and relapse phases (10). The complete distribution of all six original stages and detailed rationale for stage grouping are provided in Supplementary Appendix B. The distribution of the combined stages based on plant-based protein intake was as follows: Early stages (PC + C): 331 (83.2%); low: 141 (94.6%), moderate: 136 (82.9%), high: 54 (63.5%); Transition stages (P + R): 52 (13.1%); low: 6 (4.0%), moderate: 25 (15.2%), high: 21 (24.7%); Established adoption (A + M): 15 (3.8%); low: 2 (1.3%), moderate: 3 (1.8%), high: 10 (11.8%).

This grouping reflects the non-linear nature of behavior change and accounts for individuals who may cycle between preparation and relapse phases (10). It also aligns with the core structure of the Transtheoretical Model: early stages (PC + C) capture individuals not yet engaging in the behavior and not actively preparing to do so; transition stages (P + R) capture individuals actively involved in behavior change but in unstable states either preparing for initial adoption or having adopted and then discontinued; and established adoption (A + M) represents individuals currently practicing the behavior (A) or sustaining it over time (M), indicating stable adoption (10). Sensitivity analysis using unreduced stages was conducted to examine the robustness of this grouping approach (see Supplementary Appendix B).”

#### 2.2.4 Sustainable and healthy eating behaviors (SHEBs)

Sustainable and healthy eating behaviors were assessed using the 34-item scale developed and validated by Żakowska-Biemans et al. (31), widely applied in research on the intersection of human, animal, and environmental health. The scale includes eight components: healthy and balanced diet (10 items), regional and organic quality labels (5 items), reducing meat consumption (4 items), local foods (3 items), low-fat products (3 items), food waste (3 items), animal welfare (3 items), and seasonal food (3 items).

Participants rated their engagement in each behavior on a 7-point Likert scale ranging from 1 (“Never”) to 7 (“Always”). Component scores were calculated by averaging the items within each dimension, and the overall SHEBs score was calculated as the average across all dimensions. Higher scores indicated greater engagement in SHEBs. Reliability was high, with Cronbach’s alpha values ranging from 0.904 to 0.908 for the components and 0.909 for the total scale, consistent with prior studies (32, 33). An Arabic version was developed using forward–backward translation and piloted among 20 Saudi adults.

#### 2.2.5 Motives for choosing SHDs

Participants indicated their motivations for following SHDs by selecting one or more of eight options: health, taste, cost, environmental sustainability, animal welfare, weight management, accessibility, and enjoyment.

#### 2.2.6 Familiarity with SHDs

Participants’ familiarity with SHDs was assessed with a single item based on the FAO definition: “*Are you familiar with the FAO definition of SHDs: ‘nutritionally adequate, safe, and healthy diets that meet the nutritional needs of present and future generations, respect biodiversity and ecosystems, are protective, culturally acceptable, accessible, economically affordable, nutritionally adequate, safe and healthy’*?” Responses were categorized as familiar, unfamiliar, or partially familiar.

You may insert up to 5 heading levels into your manuscript as can be seen in “Styles” tab of this template. These formatting styles are meant as a guide, as long as the heading levels are clear, Frontiers style will be applied during typesetting.

### 2.3 Statistical analysis

Descriptive statistics summarize participant characteristics, protein consumption frequency patterns (portions/week), SHEBs, motives, stages of change, and SHD familiarity. Participants were grouped into low, moderate, and high plant-based protein consumption frequency categories based on tertile distributions of total weekly portions: Low frequency (0–7 portions/week), Moderate frequency (8–21 portions/week), and High frequency (≥22 portions/week). These categories represent relative consumption frequency patterns within our sample rather than validated nutritional intake levels.

Data quality checks were performed prior to analysis. For anthropometric data, implausible values were identified using established criteria: height <130 cm or >220 cm, weight <30 kg or >200 kg, and BMI < 12 kg/m^2^ or >60 kg/m^2^. No values meeting these extreme criteria were identified in our dataset, and therefore no anthropometric data were excluded on this basis. For dietary intake data, the pre-established upper limit of 35 portions/week (5 portions/day) for each food category served as the outlier control mechanism, as described above. This threshold was applied uniformly to all participants during data collection, preventing extreme values from entering the dataset. Additionally, we examined the distribution of total protein consumption frequency (plant-based + animal-based portions/week) to identify implausible values. Total weekly protein consumption frequency ranged from 3 to 70 portions/week (mean = 42.0 ± 16.8 portions/week). No values were excluded as outliers because our FFQ captured consumption frequency patterns of selected protein sources rather than comprehensive dietary intake. One-way ANOVA tests were used for continuous data, and chi-square tests were applied to categorical outcomes.

Binary logistic regression analyses were performed to identify predictors of moderate and high plant-based protein consumption frequency, using the low-consumption frequency group as the reference group. To minimize overfitting, the “10 events per predictor” rule (34) was applied, limiting models to 16 predictors for moderate consumption (*n* = 164) and 8 predictors for high consumption (*n* = 85). Stepwise backward elimination was employed to remove variables with *p* ≥ 0.05 or evidence of multicollinearity (variance inflation factor, VIF > 4.0). Final models retained only statistically significant predictors. Although we used portions/week as continuous variables in regression models for statistical modeling purposes, these should be interpreted as relative indicators of consumption patterns rather than validated quantitative intake measures. The odds ratios represent associations with relative differences in consumption frequency patterns.

To control Type I error inflation from multiple comparisons, Bonferroni correction was applied, adjusting the significance threshold to *p* < 0.017 (0.05/3) for pairwise comparisons. Model performance was evaluated using the area under the receiver operating characteristic curve (AUC), Nagelkerke *R*^2^, and the Hosmer-Lemeshow goodness-of-fit test. Classification accuracy was reported as the proportion of correctly classified cases. Odds ratios (ORs) with 95% confidence intervals (CIs) were presented, where OR >1 indicated increased odds and OR <1 indicated decreased odds of being classified in the higher plant-based protein consumption frequency group.

Because of multicollinearity with individual components (VIF > 4.0), the total SHEBs score was excluded from the final models. Multicollinearity diagnostics were performed for all predictors retained in final models using variance inflation factor (VIF) calculations. All predictors in the final models had VIF values below 3.5, well under the threshold of 4.0, confirming absence of problematic multicollinearity. Tolerance values (1/VIF) were all above 0.30, further supporting model stability. Statistical analyses were conducted using IBM SPSS Statistics, version 29.0 (IBM Corp., Armonk, NY, USA), with significance set at *p* < 0.05.

## 3 Results

### 3.1 Participant characteristics

A total of 398 participants in this convenience sample were included in the analysis, with a mean age of 21.22 ± 2.12 years. As a result of the snowball sampling methodology through university networks and social media platforms, the majority were female (74.1%), single (70.9%), and had attained university-level education or higher (68.8%). Approximately 63.8% reported a monthly income of SAR 10,000 or less. Gender distribution differed significantly across levels of plant-protein consumption, with a higher proportion of females in the high-intake group (*p* = 0.021). No significant differences were observed across income, marital status, or educational level (Table 1).

**Table 1:** Demographic characteristics of participant (*n* = 398).

Variable	All participants 398	Low frequency (0–7 port/wk) *n* = 149 (37.4%)	Moderate frequency (8–21 port/wk) *n* = 164 (41.2%)	High frequency (≥22 port/wk) *n* = 85 (21.4%)	*p*-value
Mean (SD) or *n* (%)					
Age	21.22 ± 2.117	21.31 ± 2. 089	21.49 ± 2.233	21.52 ± 1.777	0.051
Gender					
Male	103 (25.9)	41 (27.5)	47 (28.7)	15 (17.6)	0.021^*a^
Female	295 (74.1)	108 (72.5)	117 (71.3)	70 (82.4)	
Educational level					
Secondary school or lower	124 (31.2)	50 (33.6)	51 (31.1)	23 (27.1)	0.587^b^
University or higher	274 (68.8)	99 (66.4)	113 (68.9)	62(72.9)	
Family monthly income					
More than ^c^SR 10,000	144 (36.2)	52 (34.9)	61 (37.2)	31 (36.5)	0.913^b^
SR 10,000 or less	254 (63.8)	97 (65.1)	103 (62.8)	54 (63.5)	
Marital status					
Single	282 (70.9)	109 (73.2)	112 (68.3)	61 (71.8)	0.626^b^
Married	116 (29.1)	40 (26.8)	52 (31.7)	24 (28.2)	

^a^ ANOVA test; ^b^ Chi-Square test; *p ≤ 0.05; SD, standard deviation, ^c^ Saudi Riyal.

### 3.2 Protein source consumption frequency and stages of change

The median weekly consumption frequency of plant-based protein sources was 14 portions/week (IQR: 7–24), compared with 28 portions/week (IQR: 21–35) for animal-based protein sources. Participants in the high frequency group consumed a median of 28 plant-based protein portions/week (IQR: 24–33), compared with 4 portions/week (IQR: 2–6) in the low frequency group (Kruskal- Wallis *H* = 287.4, *p* < 0.001). Conversely, animal-based protein consumption frequency was highest in the low plant-protein group (median 30 portions/week, IQR: 24–35) and lowest in the high group (median 24 portions/week, IQR: 18–31; *H* = 24.8, *p* < 0.001).

The ratio of animal- to plant-based protein consumption frequency varied significantly across groups (*H* = 245.1, *p* < 0.001): approximately 6:1 in the low-frequency group (median 6.0, IQR: 4.0–12.0), 2:1 in the moderate group (median 2.0, IQR: 1.5–2.8), and approximately 1:1 in the high group (median 0.9, IQR: 0.7–1.2), indicating a progressive shift toward more balanced incorporation of plant-based protein sources.

Regarding readiness to adopt a plant-based diet, most participants (83.2%) were in the precontemplation or contemplation stages, 13.1% were in preparation or relapse, and only 3.8% were in action or maintenance. The distribution of stages differed significantly across consumption frequency groups (χ^2^ = 46.7, df = 4, *p* < 0.001). Notably, participants with high plant-based protein consumption frequency were more likely to be in advanced stages of change than those with low frequency, with 11.8% in action/maintenance compared to only 1.3% in the low-frequency group (Table 2).

**Table 2:** Participant consumption frequency (portions/week), stages of change, and BMI (*n* = 398).

Variable	All participants (*n* = 398)	Low frequency (0–7 port/wk) *n* = 149 (37.4%)	Moderate frequency (8–21 port/wk) *n* = 164 (41.2%)	High frequency (≥22 port/wk) *n* = 85 (21.4%)	*p*-value
Animal-based protein portions/week	28 (21–35)	30 (24–35)	28 (21–33)	24 (18–31)	<0.001***ᵃ
Plant-based protein portions/week	14 (7–24)	4 (2–6)	14 (11–18)	28 (24–33)	<0.001***ᵃ
Ratio (Animal: plant)	2.0 (1.2–4.3)	6.0 (4.0–12.0)	2.0 (1.5–2.8)	0.9 (0.7–1.2)	<0.001***ᵃ
Stages of change to adopt plant-based diet, *n* (%)					<0.001***ᵇ
PC/C	331 (83.2)	141 (94.6)	136 (82.9)	54 (63.5)	
P/R	52 (13.1)	6 (4.0)	25 (15.2)	21 (24.7)	
A/M	15 (3.8)	2 (1.3)	3 (1.8)	10 (11.8)	
Weight (kg)	60.0 (50–70)	60.0 (49–72)	61.0 (51–70)	58.0 (49–68)	0.025*ᵃ
BMI (kg/m^2^)	24.31 ± 6.50	24.57 ± 7.02	24.12 ± 5.98	23.19 ± 6.58	0.031*ᶜ
BMI categories, *n* (%)					0.017*ᵇ
Underweight	59 (14.8)	11 (7.4)	35 (21.3)	13 (15.3)	
Normal weight	187 (47.0)	83 (55.7)	64 (39.0)	40 (47.1)	
Overweight	103 (25.9)	36 (24.2)	44 (26.8)	23 (27.1)	
Obese	49 (12.3)	19 (12.8)	21 (12.8)	9 (10.6)	

Values are presented as median (IQR), mean ± SD, or n (%) as appropriate. ᵃ Kruskal–Wallis test; ᵇ Chi-square test; ᶜ ANOVA. *p ≤ 0.05; **p ≤ 0.0001. IQR, interquartile range; PC, pre-contemplation; C, contemplation; P, planning; R, relapse; A, action; M, maintenance; port/wk, portions per week. Consumption frequency represents self-reported portions per week of food categories and does not reflect validated protein intake or nutritional adequacy. Ratios represent the relative consumption frequency of animal- versus plant-based protein sources within dietary patterns and are not validated nutritional comparisons. ***p ≤ 0.0001.

### 3.3 Anthropometric characteristics

The mean body weight was 60.98 ± 17.95 kg, with an average BMI of 24.31 ± 6.50 kg/m^2^. Significant differences were observed across intake groups: the high plant-protein group reported a lower mean BMI (23.19 ± 6.58) than the low-intake group (24.57 ± 7.02, *p* = 0.031). BMI category distribution also differed significantly (*p* = 0.017), with underweight status more common among moderate consumers (21.3%), while overweight and obesity were more prevalent among low consumers (Table 2). These cross-sectional associations do not establish whether plant-protein intake influences BMI or whether individuals with lower BMI are more likely to consume plant-based proteins.

### 3.4 SHEBs

Sustainable healthy eating behaviors were positively associated with higher levels of plant-protein intake. Participants in the high-intake group scored significantly higher on indicators such as following a healthy balanced diet (5.91 ± 1.45), choosing local foods (1.01 ± 0.54), reducing meat consumption (1.86 ± 0.71), minimizing food waste (1.82 ± 0.33), and supporting animal welfare (1.27 ± 0.46), with all *p*-values < 0.001 except for low-fat products. The composite SHEBs score increased progressively across intake categories, from 1.91 ± 0.38 in the low group to 2.17 ± 0.43 in the high group (*p* < 0.001) (Table 3).

**Table 3:** Participants’ SHEBs based on daily plant-based protein consumption (*n* = 398).

Variable	All participants 398	Low Frequency (0–7 port/wk) *n* = 149 (37.4%)	Moderate frequency (8–21 port/wk) *n* = 164 (41.2%)	High frequency (≥22 port/wk) *n* = 85 (21.4%)	*P*-value^a^
Mean (SD) or *n* (%)					
Healthy balanced diet	5.62 ± 1.48	5.34 ± 1.53	5.72 ± 1.42	**5**.91 ± 1.45	0.010^*a^
Quality labels	2.19 ± 0.88	1.97 ± 0 0.96	2.29 ± 0.86	2.37 ± 0.68	0.001^* a^
Meat reduction	1.64 ± 0 0.62	1.51 ± 0.58	1.66 ± 0.57	1.86 ± 0 0.71	0.000^** a^
Local foods	0.91 ± 0 0.48	0.65 ± 0.42	0.86 ± 0.49	1.01 ± 0.54	0.000^** a^
Low fat	1.10 ± 0.57	1.04 ± 0 0.57	1.11 ± 0.56	1.21 ± 0.53	0.164
Food waste	1.73 ± 0 0.41	1.60 ± 0 0.49	1.72 ± 0.39	1.82 ± 0.33	0.000^** a^
Animal welfare	1.12 ± 0 0.47	1.01 ± 0 0.47	1.14 ± 0 0.44	1.27 ± 0.46	0.000^** a^
Seasonal food	0.95 ± 0.43	0.79 ± 0 0.41	1.00 ± 0.40	1.23 ± 0.44	0.000^** a^
Total SHEBs	2.03 ± 0.42	1.91 ± 0.38	2.07 ± 0 0.42	2.17 ± 0.43	0.000^** a^
Familiarity with a sustainable healthy diet					
Familiar	94 (23.6)	25 (16.8)	44 (26.8)	25 (29.4)	0.001*^b^
Unfamiliar	172 (43.2)	85 (57)	56 (34.1)	31 (36.5)	
Partial familiarity	132 (33.2)	39 (26.2)	64 (39)	29 (34.1)	

^a^ ANOVA test; ^b^ Chi-Square test; *P ≤ 0.05; **P ≤ 0.001; ***P ≤ 0.0001; SD, standard deviation.

### 3.5 Familiarity with SHDs

When asked about the FAO definition of SHDs, 23.6% of participants reported being unfamiliar, and 33.2% indicated they were partially familiar. Familiarity differed significantly across intake groups (*p* = 0.001), with greater familiarity observed among participants in the moderate and high plant-based protein categories (Table 3).

### 3.6 Motives for choosing SHDs

**Figure 1:**
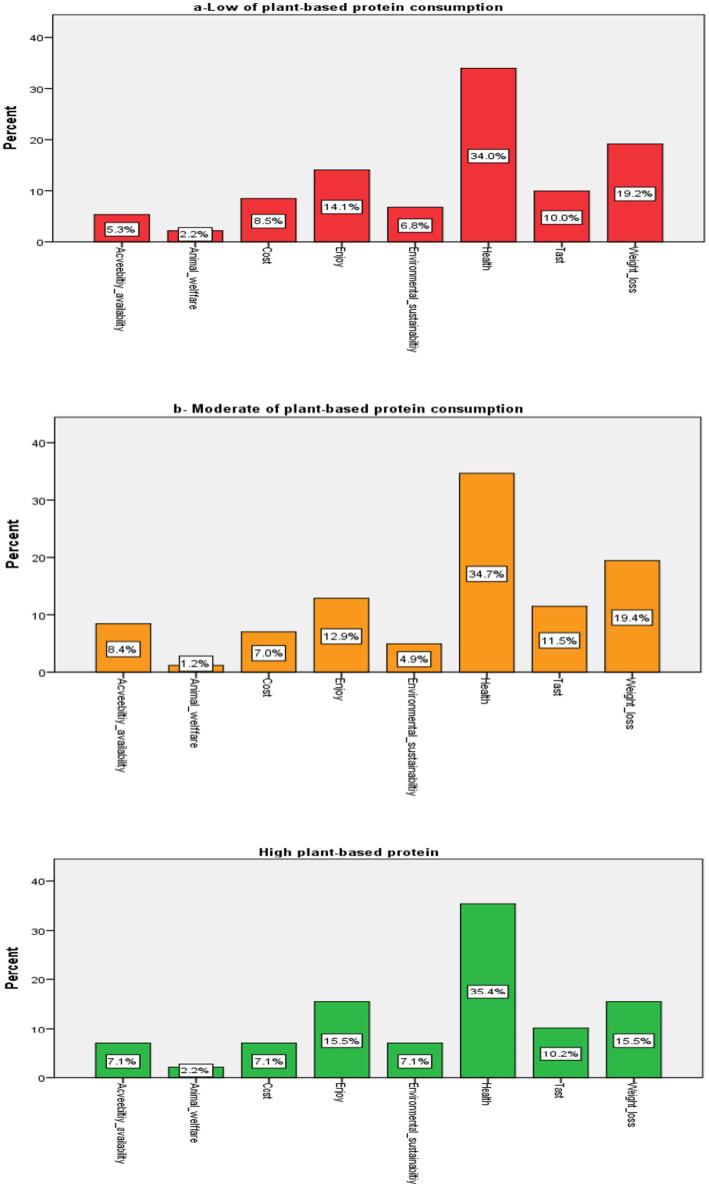
Motivations for consuming plant-based protein.

Analysis of participants’ motivations for choosing SHDs revealed differences between high and low-consumption groups. Across all participants, health emerged as the primary motivator, reported by more than 35%. Among those with low and moderate plant-based protein intake, weight management was the second most frequently cited motivation (19%), followed by enjoyment. In the high-intake group, weight management and enjoyment were equally ranked as the second motivators (15.5% each). Taste consistently ranked fourth across all groups. For environmental sustainability, the highest percentage was reported by the high-intake group (7.1%), compared with 6.8% in the low-intake group and 4.9% in the moderate-intake group. Animal welfare consistently ranked last across all groups. Importantly, no statistically significant differences were observed between groups for any of the motivations (Figure 1). Therefore, while descriptive patterns suggest possible variation in motivation profiles across consumption groups, these differences cannot be interpreted as statistically meaningful given the non-significant *p*-values. The primary finding is that health motivations predominate across all consumption levels.

Binary logistic regression analysis identified seven significant predictors of moderate versus low plant-based protein consumption frequency (Table 4). The model demonstrated strong discriminatory ability (AUC = 0.79, 95% CI: 0.74–0.85) and explained 38.7% of the variance (Nagelkerke *R*^2^ = 0.387). Model fit was adequate (Hosmer–Lemeshow χ^2^ = 11.24, *p* = 0.189), with an overall classification accuracy of 74.1%. Among anthropometric variables, participants with normal BMI had three times higher odds of moderate plant-protein consumption frequency than underweight individuals (OR = 3.10, 95% CI: 1.21–7.93, *p* = 0.018). Among sustainable healthy eating behaviors, seasonal food consumption showed the strongest association (OR = 3.10, 95% CI: 1.73–5.54, *p* < 0.001), meaning each 1-point increase on the 7-point seasonal food scale was associated with 3.1 times higher odds of moderate consumption frequency. Local food consumption also showed a strong positive association (OR = 2.54, 95% CI: 1.52–4.26, p < 0.001). Quality-label consideration (OR = 1.40, 95% CI: 1.07–1.84, *p* = 0.013) and adherence to a healthy, balanced diet (OR = 1.19, 95% CI: 1.02–1.38, *p* = 0.026) were positively associated with moderate consumption frequency, though with smaller effect sizes. Partial familiarity with SHDs was associated with significantly lower odds of moderate plant-protein consumption compared to being unfamiliar (OR = 0.39, 95% CI: 0.23–0.65, *p* = 0.003), suggesting that incomplete knowledge may act as a barrier to adoption. Food waste concerns showed an inverse relationship (OR = 0.38, 95% CI: 0.20–0.73, p = 0.003). All variance inflation factors were below 2.5, confirming absence of problematic multicollinearity among retained predictors (Table 4).

**Table 4:** Predictors of moderate vs. low plant-based protein consumption frequency (*n* = 313).

Variables	OR	95% Confidence interval (CI)	*P*-value	VIF
Anthropometric variables				
Normal weight (vs. underweight)	3.10	1.21–7.93	0.018*	1.18
SHEBs				
Healthy balanced diet (per 1-point increase)	1.19	1.02–1.38	0.026*	2.31
Quality labels (per 1-point increase)	1.40	1.07–1.84	0.013*	1.67
Local foods (per 1-point increase)	2.54	1.52–4.26	<0.001***	2.18
Food waste (per 1-point increase)	0.38	0.20–0.73	0.003*	1.89
Seasonal food (per 1-point increase)	3.10	1.73–5.54	<0.001***	2.43
SHD familiarity				
Partial familiarity (vs. unfamiliar)	0.39	0.23–0.65	0.003*	1.34
Model performance:	AUC = 0.79 (95% CI: 0.74–0.85) Nagelkerke R^2^ = 0.387 (38.7%) Hosmer–Lemeshow χ^2^ = 11.24, *p* = 0.189 (good fit) Classification accuracy = 74.1% Events per predictor = 23.4 − 2 Log Likelihood = 345.2			

*P ≤ 0.05; **P ≤ 0.0001. The dependent variable is binary (moderate vs. low consumption frequency). OR >1 indicates higher odds of being in the moderate consumption group. Total SHEBs score was excluded due to multicollinearity with individual components (VIF >4.0). All retained predictors had VIF <3.0. SHEBs were measured using 7-point Likert scales (1 = Never, 7 = Always).

Binary logistic regression analysis identified eight significant predictors of high versus low plant-based protein consumption frequency (Table 5). The model demonstrated strong discriminatory ability (AUC = 0.87, 95% CI: 0.82--0.92) and explained 52.1% of the variance (Nagelkerke *R*^2^ = 0.521). Model fit was adequate (Hosmer--Lemeshow χ^2^ = 6.78, *p* = 0.561), with an overall classification accuracy of 81.6%. Among stages of change variables, participants in the preparation/relapse stage had significantly reduced odds of high plant-based protein consumption frequency compared to those in precontemplation/contemplation stages (OR = 0.12, 95% CI: 0.03–0.48, *p* = 0.003). Regarding animal-based protein consumption patterns, each increase of 7 portions/week in animal-based protein frequency was associated with 15% lower odds of high plant-based protein consumption frequency (OR = 0.85, 95% CI: 0.79–0.91, *p* < 0.001).

**Table 5:** Predictors of high vs. low plant-based protein consumption frequency (*n* = 234).

Variables	OR	95% Confidence interval (CI)	P-value	VIF
Stages of change				
P/R stage (vs. PC/C)	0.12	0.03–0.48	0.003*	1.23
Animal-based protein consumption pattern				
Animal protein frequency (per 7 portions/week increase)	0.85	0.79–0.91	<0.001***	1.45
SHEBs				
Healthy balanced diet (per 1-point increase)	1.41	1.12–1.78	0.004*	2.67
Meat reduction behaviors (per 1-point increase)	2.41	1.35–4.30	0.003*	2.89
Local foods (per 1-point increase)	5.12	2.58–10.21	<0.001***	2.76
Food waste (per 1-point increase)	0.18	0.07–0.45	<0.001***	1.98
Animal welfare (per 1-point increase)	3.67	1.72–7.83	0.001**	2.34
Seasonal food (per 1-point increase)	7.89	3.42–18.20	<0.001***	2.91
Model performance:	AUC = 0.87 (95% CI: 0.82–0.92) Nagelkerke *R*^2^ = 0.521 (52.1%) Hosmer–Lemeshow χ^2^ = 6.78, *p* = 0.561 (good fit). Classification accuracy = 81.6% Events per predictor = 10.6 − 2 Log Likelihood = 178.4			

*P ≤ 0.05; **P ≤ 0.001; **P ≤ 0.0001. The dependent variable is binary (high vs. low consumption frequency). Higher animal-based protein consumption frequency (per 7 portions/week increase) was associated with 15% lower odds of high plant-based protein consumption (OR = 0.85), reflecting dietary substitution patterns. All VIF values were <3.0, indicating no problematic multicollinearity. SHEBs were measured using 7-point Likert scales (1 = Never, 7 = Always).

Among sustainable healthy eating behaviors, seasonal food consumption showed the strongest association (OR = 7.89, 95% CI: 3.42–18.20, *p* < 0.001). Local food consumption also showed a strong positive association (OR = 5.12, 95% CI: 2.58–10.21, *p* < 0.001). Animal welfare considerations (OR = 3.67, 95% CI: 1.72–7.83, *p* = 0.001), meat-reduction behaviors (OR = 2.41, 95% CI: 1.35–4.30, *p* = 0.003), and adherence to a healthy, balanced diet (OR = 1.41, 95% CI: 1.12–1.78, *p* = 0.004) were positively associated with high consumption frequency. Food waste concerns showed a significant inverse relationship (OR = 0.18, 95% CI: 0.07–0.45, *p* < 0.001). All variance inflation factors were below 3.0, confirming absence of problematic multicollinearity among retained predictors (Table 5).

## 4 Discussion

This cross-sectional study provides a descriptive examination of plant-based protein consumption frequency patterns among a convenience sample of Saudi Gen Z, documenting co-occurrence patterns between consumption frequencies of plant-based protein sources, sustainable healthy eating behaviors (SHEBs), and health outcomes within this culturally unique population. The findings add to the limited research on sustainable dietary patterns in the Gulf region and highlight both opportunities and barriers for promoting plant-forward diets among young Saudi adults.

The predominance of participants in low consumption frequency categories suggests limited incorporation of these food sources into regular meal patterns within our sample. However, without validated quantitative intake data, direct comparisons to consumption patterns in other populations cannot be made.

Several interrelated factors may explain the low consumption frequency observed in our study. Western populations benefit from decades of public health campaigns promoting legumes, whole grains, and plant-based proteins for cardiovascular and metabolic health, coupled with strong environmental advocacy supporting SHDs. Furthermore, plant-based alternatives such as soy products, fortified plant milks, and meat analogs are widely available and affordable, facilitating dietary substitutions (35).

By contrast, Saudi and Gulf diets remain meat-centric, with animal protein playing a central cultural role in hospitality, social gatherings, and celebrations (20, 21). In Saudi culture, meat holds deep symbolic significance, representing generosity, social status, and celebration (19), and this cultural embeddedness may limit the adoption of plant-protein diets, even among environmentally conscious youth (36). Although traditional Saudi cuisine does feature plant protein sources such as lentils, chickpeas, and fava beans, these foods have declined in prominence during the ongoing dietary transition toward more Westernized eating patterns. This shift has resulted in both a reduced reliance on traditional plant-rich dishes and an increased dependence on animal proteins. The lack of movement away from traditional plant-based products further exacerbates this trend. Collectively, these factors help explain the notably low consumption frequency of plant-protein sources observed among Saudi Gen Z. As Rothgerber (18) emphasizes, cultural identity strongly intersects with dietary behavior, often outweighing health or environmental motivations. Therefore, strategies to increase plant protein consumption in Saudi Arabia must address these cultural dimensions while promoting nutritionally adequate, culturally acceptable plant-based alternatives that can coexist with, rather than replace, traditional food practices.

Nevertheless, 21.4% of participants in our sample achieved high consumption frequency (≥22 portions/week) of plant-based protein sources, indicating some degree of emerging dietary diversification despite the cultural emphasis on meat-based hospitality and traditional food practices in Saudi Arabia. However, several factors limit interpretation of this finding. Our convenience sample, comprising predominantly female (74%) and university-educated (69%) participants recruited through social media, likely overrepresents health-conscious individuals with greater exposure to nutrition information and plant-based options. Consequently, this proportion may not reflect prevalence in the broader Saudi Gen Z population. Additionally, our cross-sectional design cannot determine whether these consumption patterns represent stable dietary behaviors or temporary fluctuations. Whether these individuals represent genuine early adopters, whether their behaviors remain stable over time, and whether their dietary choices influence others in their social networks are empirical questions requiring representative longitudinal research to address. This finding suggests that a notable minority of young Saudis are beginning to diversify their protein sources, potentially representing early adopters of SHDs pattern. Importantly, even among high-frequency consumers, the median consumption of 28 plant-based protein portions/week remains substantially lower than the 28 portions/week observed for animal-based protein sources across all participants, underscoring the continued dominance of animal proteins in dietary patterns.

Recent studies from Kuwait (22) and the United Arab Emirates (23, 24) similarly report rising environmental and health awareness among young adults. Yet, adoption of plant-forward diets remains limited across the Gulf region. These regional findings align with our results and suggest that while environmental consciousness is growing among Gulf youth, translating awareness into sustained dietary behavior change remains challenging due to deeply embedded cultural food practices. The Saudi context is somewhat distinct: persistent cultural barriers to vegetarianism coexist with a measurable minority achieving higher plant-protein consumption frequency. This pattern suggests that while meat retains strong symbolic and cultural significance (19), targeted interventions could support gradual dietary shifts among younger Saudis who appear increasingly receptive to alternative protein sources.

The inverse relationship observed between plant- and animal-protein consumption frequency across intake groups suggests substitution patterns within our sample’s dietary behaviors. In this study, the progression from a 6:1 animal-to-plant protein consumption frequency ratio in the low-frequency group to an approximately 1:1 ratio in the high-frequency group suggests a gradual move toward more balanced incorporation of plant-based protein sources into meal patterns.

However, this shift in Saudi Arabia occurs against the backdrop of longstanding cultural traditions in which meat holds deep symbolic significance, representing generosity, social status, and celebration (19). Such cultural embeddedness may limit the adoption of plant-protein diets, even among environmentally conscious youth (36). As Rothgerber (18) emphasizes, cultural identity strongly intersects with dietary behavior, often outweighing health or environmental motivations. Therefore, strategies to increase plant protein consumption in Saudi Arabia must address these cultural dimensions while promoting nutritionally adequate, culturally acceptable plant-based alternatives.

Consistent with previous research, women in our study reported significantly higher plant protein consumption frequency than men (10, 37). However, gender was not a significant independent predictor in the logistic regression model, suggesting that once behavioral, sustainability, and dietary factors were controlled for, gender differences diminished. This aligns with Hruby and Jacques (7), who found that health- and weight-related motivations often transcend gender when predicting dietary behaviors. Similarly, in the present study, weight management emerged as the second most common motivation for higher plant-protein consumption frequency.

The association between plant-protein consumption frequency and body weight revealed important but nuanced patterns. Although the highest proportion of participants with normal BMI was found in the low-consumption frequency group (55.7%, vs. 39.0% in the moderate group and 47.1% in the high group), the mean BMI was lowest among high-consumption frequency participants (23.19 ± 6.58 vs. 24.57 ± 7.02 kg/m^2^ in the low group). Moreover, the prevalence of overweight and obesity was slightly lower among high plant-protein frequency consumers than among those with low consumption frequency. These cross-sectional associations align with earlier research observing relationships between plant-forward diets and healthier weight outcomes (7, 20). However, our study design precludes causal inference we cannot determine whether higher consumption frequency of plant-based protein sources is associated with lower BMI through direct effects on weight, or whether individuals with lower BMI are more likely to adopt dietary patterns that include plant-based protein sources more frequently, or whether both are influenced by unmeasured third variables such as overall health consciousness. Longitudinal and intervention studies are needed to establish temporal relationships and evaluate whether interventions targeting plant-protein consumption frequency might influence weight outcomes in the context of Saudi Arabia’s obesity epidemic, where more than 60% of the population is under 30 years old (13).

Regression analysis revealed that normal weight status was associated with higher odds of moderate plant-protein consumption frequency (OR = 3.10), reflecting a complex pattern that may indicate bidirectional associations, reverse causation (health status influencing dietary choices), or confounding by unmeasured variables. A similar pattern was reported among Kuwait university students, where those with normal BMI were 1.9 times more likely to have positive attitudes toward SHDs compared with obese peers (22). Several potential explanations exist for this association. Higher consumption frequency of plant-protein sources may co-occur with weight management through mechanisms such as dietary patterns that include higher fiber content, lower energy density, and improved satiety (4, 18). Alternatively, individuals who maintain healthy body weight may be more health-conscious overall, adopting multiple beneficial lifestyle behaviors simultaneously—a well-documented phenomenon in the public health literature (38). However, our cross-sectional design cannot determine the direction of causality or distinguish between these competing explanations.

Interestingly, findings from other populations indicate that this association is not always linear. For example, a study among Indonesian women found that higher plant-protein intake was significantly associated with higher BMI after adjusting for marital status and age (*p* = 0.043; *R*^2^ = 0.080), emphasizing the importance of protein quality and overall dietary context when interpreting links between plant-protein intake and obesity among women of reproductive age (39).

The observation that low plant-protein frequency consumers had the highest proportion of normal BMI likely reflects broader lifestyle and dietary factors rather than plant-protein consumption alone. For example, individuals with lower plant-protein consumption frequency may also have reduced total energy intake, different meal compositions, or higher physical activity levels, all of which could influence BMI independent of protein source consumption frequency. Conversely, participants in the high plant-protein frequency group may consume energy-dense plant foods such as legumes, grains, and nuts more frequently. Although these foods are nutrient-rich, frequent consumption could contribute to higher BMI if total caloric consumption exceeds energy expenditure. Such complex interactions underscore the need for longitudinal studies to disentangle temporal relationships and potential causal pathways and inform culturally appropriate dietary interventions for weight management and chronic disease prevention in Saudi Arabia, where weight control is a primary motivator for adopting SHDs that emphasize plant protein.

A noteworthy finding is that 83.2% of participants remained in the precontemplation or contemplation stages regarding plant-based diet adoption, indicating a substantial opportunity for tailored behavioral interventions. This distribution of readiness aligns with established patterns observed in other applications of the stages of change to dietary behaviors (11) and underscores the necessity for stage-matched strategies, such as targeted awareness campaigns for early stages and skill-building workshops for those in preparation. Importantly, a positive association was observed between higher plant-protein consumption frequency and more advanced stages of change (*p* < 0.001). Our cross-sectional design cannot determine whether more frequent exposure to plant-protein foods catalyzes behavioral progression, whether individuals in advanced stages increase their consumption frequency, or whether both reflect underlying motivational factors. The association admits multiple interpretations.

However, the paradoxical finding that participants in the preparation/relapse stage had significantly reduced odds of high plant-protein consumption frequency (OR = 0.121) merits careful interpretation. This counterintuitive result likely reflects the cyclical and non-linear nature of dietary change, in which individuals in transition often experience fluctuating consumption frequency and relapse before achieving sustained behaviors (10, 11). Consequently, these results highlight the critical importance of continuous, culturally relevant reinforcement and sustained support mechanisms during transitional stages to prevent relapses and facilitate successful progression to maintenance. A detailed examination of this counterintuitive finding in relation to the observed data distribution is provided in Supplementary Appendix B.

Sustainable healthy eating behaviors were the strongest predictors of both high and moderate plant-protein consumption frequency in our regression models, consistent with the interpretation that plant-protein consumption frequency co-occurs with broader sustainable eating patterns rather than occurring in isolation. Specifically, in our sample, participants with high plant-protein consumption frequency showed particularly strong associations with meat-reduction behaviors (OR = 2.41), consumption of locally produced food (OR = 5.12; moderate group OR = 2.54), seasonal food choices (OR = 7.89; moderate group OR = 3.10), and animal welfare considerations (OR = 3.67) compared to low consumers. The particularly strong association observed for seasonal food consumption (OR = 7.89) warrants contextual interpretation. This large effect size likely reflects the fact that prioritizing seasonal foods represents a comprehensive sustainable eating orientation rather than a single isolated behavior. Individuals who consistently choose seasonal foods demonstrate commitment to local food systems, environmental sustainability, and dietary planning—all characteristics that strongly align with plant-based eating patterns. Similar magnitudes of association have been reported in studies examining relationships between integrated lifestyle behaviors and dietary patterns (31, 32). Together, these findings reflect a coherent, value-driven approach to food selection that extends beyond protein-source preferences.

These associations indicate that, among our participants, higher plant-protein consumption frequency co-occurs with engagement in a wider range of sustainable eating practices. This aligns with Meixner et al. (40), who found that Gen Z consumers place high value on locally sourced plant-based products, consistent with the strong associations we observed with local and seasonal food choices. Similarly, Nakavachara et al. (41) reported that young consumers increasingly consider animal welfare in food decisions, aligning with our observation among high plant-protein frequency consumers (OR = 3.67).

The positive association with quality-label consideration (OR = 5.12; moderate group OR = 1.40) in our regression models suggests that among our participants, individuals who pay attention to production methods and sourcing practices are more likely to consume moderate to high frequencies of plant-based protein. This suggests that these individuals make informed choices based on production methods and sourcing practices, consistent with the heightened environmental awareness characteristic of Gen Z (8, 9). Kabaja et al. (42) also demonstrated that environmental labeling strongly shapes purchasing and consumption behaviors among Gen Z who are both highly aware of environmental issues and motivated to make eco-friendly purchasing decisions. Taken together, these findings suggest that among our sample, individuals who engage in multiple sustainable eating behaviors are more likely to consume higher frequencies of plant-based protein. Whether these individuals might influence broader dietary patterns within their social networks requires investigation through social network analysis and longitudinal observation of dietary diffusion patterns.

Unexpectedly, however, participants reporting greater concerns about food waste had lower odds of plant-protein consumption frequency in our models (moderate group OR = 0.38; high group OR = 0.18). This contrasts with findings from Europe and North America, where food-waste reduction typically accompanies sustainable dietary practices (32, 33). A plausible explanation lies in Saudi cultural norms of abundance and hospitality, including large portions, generous offerings at social events (e.g., weddings, Ramadan gatherings), and expectations to avoid scarcity. Participants may perceive plant-based proteins such as fresh legumes, plant-based milks, nuts, and produce-heavy items as more perishable than animal proteins, which are often frozen or preserved. While cultural norms of abundance may generally influence food behaviors, they do not fully explain why this pattern occurs specifically for plant-based proteins. These norms may render waste-reduction efforts incompatible with social norms of generosity. Empirical studies confirm that Saudi Arabia has high rates of household and hospitality-sector food waste, and that food-consumption culture strongly shapes waste behaviors. Even when young adults express sustainability awareness and support waste reduction policies, social expectations of abundance often prevail (43, 44).

Health motivations predominated across all consumption frequency groups (>35%), while environmental concerns remained minimal across groups. Although descriptive data suggested slightly higher environmental motivation among high plant-protein frequency consumers (7.1% vs. 6.8% in low consumers and 4.9% in moderate consumers), these differences were not statistically significant and therefore cannot support meaningful interpretation. We acknowledge this as a limitation of our categorical measurement approach and the distribution of responses, which may have limited statistical power to detect group differences.

The overall predominance of health motivations reflects global trends in which personal health benefits typically outweigh ecological considerations as primary dietary drivers (4). Future research should employ continuous measurement scales or larger sample sizes to more robustly examine whether environmental awareness differs across consumption frequency groups and whether it supports maintenance of plant-based dietary behaviors.

The observed predominance of health motivations suggests that if future longitudinal or experimental studies confirm that emphasizing health benefits increases plant-protein consumption frequency, public health interventions promoting plant-protein consumption frequency in Saudi Arabia should emphasize health benefits as the core message, while gradually incorporating environmental and sustainability themes as secondary motivators. However, our cross-sectional data cannot demonstrate that motivation-targeted messaging would be effective. Given the consistently low prioritization of environmental concerns across all groups in our sample, gradually incorporating environmental and sustainability themes as secondary motivators may be appropriate once health-based messaging is established, though further research is needed to identify the most effective messaging strategies for this population.

The consistently low prioritization of animal welfare concerns across groups may reflect cultural and religious perspectives within Islamic contexts, where humane treatment of animals is emphasized but animal consumption remains socially and religiously accepted. Furthermore, the finding that partial familiarity with SHDs predicted lower plant protein consumption frequency suggests that incomplete or superficial knowledge may impede dietary transitions. This counterintuitive result highlights the importance of nutrition education programs that provide comprehensive, actionable guidance rather than superficial awareness-raising activities.

This study has several notable strengths. It represents the first comprehensive assessment of plant-protein consumption frequency patterns among Gen Z in Saudi Arabia, offering a unique contribution to the limited literature on sustainable dietary practices in the Gulf region. By integrating dietary consumption frequency data, anthropometric measures, behavioral motivations, and stages of change, the study provides a multidimensional perspective on factors influencing plant-protein adoption. The use of validated and culturally adapted instruments enhances reliability and contextual relevance, while the relatively large sample size (*n* = 398) strengthens statistical robustness. Additionally, the identification of behavioral predictors, such as sustainable eating habits and local food preferences, offers valuable insights for designing targeted interventions to promote sustainable and healthy dietary transitions among young Saudi adults.

Despite its strengths, several limitations should be acknowledged. First, the cross-sectional design precludes causal inferences, limiting the ability to establish temporal relationships between plant-protein consumption frequency, health outcomes, and behavioral predictors. All observed associations should be interpreted as correlational rather than causal, and the directionality of these relationships remains uncertain. For example, we cannot determine whether higher plant-protein consumption frequency leads to lower BMI or whether individuals with lower BMI are more likely to adopt dietary patterns that incorporate plant-based protein sources more frequently. Second, the consumption frequency estimates derived from our adapted FFQ represent relative consumption frequency patterns within our sample rather than validated measures of absolute protein intake or nutritional adequacy. Our FFQ captured consumption frequency of selected plant protein sources (legumes, nuts, plant-based alternatives) rather than comprehensive dietary intake, excluding incidental plant proteins from grains, vegetables, and mixed dishes that contribute substantially to total consumption. Consequently, our findings should be interpreted as reflecting relative differences in consumption frequency patterns across groups rather than absolute nutritional intake levels. While this limitation affects the ability to make nutritional adequacy assessments, it does not invalidate the observed associations between relative consumption frequency levels and health outcomes, sustainable eating behaviors, and dietary readiness. Our use of consumption frequency values in ratios and as continuous predictors in regression models represents a methodological approach to pattern analysis rather than validated quantitative assessment.

Third, reliance on self-reported dietary consumption frequency and anthropometric measures introduces potential recall and reporting biases, which may affect the accuracy of dietary estimates and BMI classification. Self-reported weight and height are known to be subject to social desirability bias, with individuals typically underreporting weight and overreporting height, leading to underestimation of BMI by 0.5–2.0 kg/m^2^ in validation studies (45). This systematic bias may have resulted in misclassification of BMI categories, particularly underestimation of overweight and obesity prevalence in our sample. The direction of bias (underestimation) suggests that actual BMI values may be higher than reported, potentially affecting the observed associations between plant-protein consumption frequency and BMI categories. Similarly, self-reported dietary consumption frequency through FFQ is subject to recall bias, estimation errors, and social desirability bias, which may have affected the accuracy of consumption frequency estimates (46).

Fourth, the use of convenience and snowball sampling through university networks and WhatsApp limits the generalizability of the findings to the broader Saudi Gen Z population because the sample does not reflect key demographic and socioeconomic characteristics of that population. Women made up 74.1% of participants far above the national gender distribution which may lead to inflated estimates of plant-based protein consumption frequency and sustainable eating interest. Similarly, 68.8% of respondents had university-level education or higher, meaning the sample mostly represents academically engaged individuals who typically show greater health and nutrition awareness. The online format also required reliable smartphone and internet access, unintentionally excluding lower-income youth and rural residents who may face practical barriers to accessing diverse food options.

Fifth, the absence of biomarker validation for dietary consumption frequency and the underrepresentation of male participants further limit external validity. Sixth, the stage combining between P and R stages may obscure meaningful differences between individuals who are preparing to adopt plant-based diets and those who have reverted after previous attempts. Likewise, grouping PC with C stages merges individuals with no intention to change with those who are already considering change, which may mask important variations in readiness for behavior change. Finally, because participation was voluntary, the survey may have primarily attracted individuals already interested in nutrition and sustainability, resulting in higher reported willingness to adopt plant-based eating patterns than would be expected in the wider population. Future research employing longitudinal or experimental designs with more representative sampling strategies and objective dietary measures would help overcome these limitations and establish causal relationships between plant-protein consumption frequency and health outcomes.

## 5 Conclusion

This study provides preliminary examination of plant-protein consumption frequency patterns among a convenience sample of young, educated, predominantly female Saudi adults, revealing low consumption frequency of plant-based protein sources relative to animal-based sources within this sample. Most participants reported being in early stages of readiness to adopt plant-forward diets. The relative differences in consumption frequency patterns across groups provide valuable insights into co-occurrence patterns among dietary behaviors and health characteristics and readiness for sustainable dietary transitions in this specific sample. Within our sample, higher plant-protein consumption frequency was associated with more favorable BMI profiles, greater engagement in SHEBs, and stronger consideration of local and seasonal foods. However, the non-representative sampling, cross-sectional design, and inability to establish causation substantially limit generalizability. These findings suggest potential research directions but cannot support intervention recommendations or population-level conclusions about Saudi Gen Z.

Future studies should employ longitudinal and intervention-based designs to establish causal relationships and evaluate the effectiveness of culturally tailored strategies to promote plant-protein consumption frequency. Attention is needed to address cultural barriers, enhance familiarity with SHDs, and integrate environmental awareness into public health messaging. Additionally, studies using more representative sampling and objective dietary assessment methods are essential to capture population-level trends and inform policy development aimed at improving both dietary sustainability and health outcomes in Saudi Arabia.

## Data Availability

The raw data supporting the conclusions of this article will be made available by the authors, without undue reservation.
