# Supplementary material for: Plant-based protein consumption patterns among Saudi Generation Z: a cross-sectional study of dietary frequencies, health outcomes, and sustainable eating behaviors
Source: Front Public Health. 2026 Jan 20;14:1739641. doi: 10.3389/fpubh.2026.1739641 (PMC12864068; doi:10.3389/fpubh.2026.1739641)
Supplement: Supplementary file 1 [file Data_Sheet_1.pdf]

Supplementary Appendix A

Food Frequency Questionnaire (FFQ) Instrument and Methodology

Plant-Based Protein Consumption Patterns Among Saudi Generation Z: A Cross-Sectional Study of Dietary Frequencies, Health Outcomes, and Sustainable Eating Behaviors: Dietary Assessment Methods

Table of Contents

1. Complete FFQ Instrument.
2. Frequency Response Options and Coding.
3. Portion Size Reference Standards for Frequency Reporting.
4. Data Quality Procedures.
5. Key Limitations.
6. References.

1. Complete FFQ Instrument

Instructions Provided to Participants (English Translation from Arabic)

"The following questions assess your usual frequency of consumption of selected foods. Please consider your typical eating habits over the past month. Report only foods you usually consume, and for each item, select the response category that best reflects your habitual intake."

Definition provided: *"One portion/serving refers to the typical amount you would eat at one time."*

Section A: Plant-Based Protein Sources

Instructions: "How often do you consume the following plant-based protein sources?"

Food Item	Description/Examples	Standard Portion
1. Cooked Legumes	Lentils, chickpeas, fava beans, kidney beans, black beans, white beans, green peas	~200g
2. Raw Nuts and Seeds	Almonds, walnuts, peanuts, pistachios, cashews, pumpkin seeds, sunflower seeds, sesame seeds	~30g
3. Plant-Based Meat Substitutes	Veggie burgers, plant-based nuggets, falafel, vegetarian meatballs	~100g
4. Plant-Based Milk	Soy milk, almond milk, oat milk, coconut milk	~240ml

Section B: Animal-Based Protein Sources

Instructions: "How often do you consume the following animal-based protein sources?"

Food Item	Description/Examples	Standard Portion
5. Red Meat	Beef, lamb, camel meat, goat	~85g
6. Poultry	Chicken, turkey	~85g
7. Fish and Seafood	Fish, shrimp, other seafood	~85g
8. Eggs	Chicken eggs	1 large egg (~50g)
9. Dairy Products	Milk, yogurt, cheese	~240ml for liquids, ~30g for cheese

Frequency Options for Each Item:

- Four or more times per day.
- Three times per day.
- Twice per day.
- Once per day.
- Five to six times per week.
- Two to four times per week.
- Once per week.
- Rarely or never.

2. Frequency Response Options and Coding

Conversion from frequency categories to portions per week

Response Option	Portions/Day	Portions/Week	Coding Rationale
Four or more times per day	4.0	28.0	Capped at upper threshold
Three times per day	3.0	21.0	Standard conversion
Twice per day	2.0	14.0	Standard conversion
Once per day	1.0	7.0	Standard conversion
Five to six times per week	0.786	5.5	Midpoint: (5+6)/2 = 5.5
Two to four times per week	0.429	3.0	Midpoint: (2+4)/2 = 3.0
Once per week	0.143	1.0	Standard conversion
Rarely or never	0	0	No consumption

Application for Upper Limit (Capping)

Rule Applied: Maximum of 5 portions per day (35 portions per week) for any single food category

Justification:

- Values exceeding 5 portions/day likely reflect reporting errors (e.g., portion size misinterpretation, recall bias) rather than actual consumption (Subar et al., 2015)
- Establishing predefined upper thresholds is a widely accepted data management strategy in nutritional epidemiology to prevent extreme values from distorting statistical analyses—an approach known as "capping" (Willett, 2013)
- This approach follows the precedent of Hagmann et al. (2019), who applied an identical 35 portions/week limit in the Swiss Food Panel study

Implementation:

- If calculated portions/week > 35 for any category, reset to 35.
- Applied to each food category independently.
- Documented in data cleaning log.

Important Note: The 35 portions/week cap is applied to EACH food category SEPARATELY. For example, if a participant reports "four or more times per day" for dairy (28 portions/week), this is within the cap. However, if they reported an implausible frequency exceeding 35 portions/week, this would be capped at 35 for that category only. Other food categories remain unaffected. This means total weekly **consumption frequency** can still be substantial if multiple categories are consumed at high (but valid) frequencies.

3. Portion Size Reference Standards for Frequency Reporting

Food Category	Standard Portion	Weight/Volume
Cooked Legumes	1 serving	~200g
Nuts and Seeds	1 serving	~30g
Plant-Based Meat Substitutes	1 piece/patty	~100g
Plant-Based Milk	1 cup	~240ml
Red Meat	1 serving	~85g
Poultry	1 serving	~85g
Fish/Seafood	1 serving	~85g
Eggs	1 large egg	~50g
Dairy	1 cup / 1 slice	~240ml for liquids, ~30g for cheese

Important:

1. These portion sizes are provided as reference standards to help participants report consumption frequency consistently. They are not used to calculate protein intake in grams. The study analysis uses only the frequency data (portions/week), not the weight/volume values listed below.
2. Portion sizes were based on established guidelines from Hagmann et al. (2019), who used standardized servings validated in the Swiss Food Panel study. These portion sizes represent typical consumption amounts for each food category.

4. Consumption Frequency Calculation Methodology

Step-by-Step Calculation Process

1. Convert frequency response to portions per week (using Section 2 conversion table)

2. Apply upper limit cap: If portions/week > 35 for any category, reset to 35

3. Sum portions across plant-based categories: Total plant-based portions/week = Cooked Legumes + Nuts/Seeds + Plant-Based Meat Substitutes + Plant-Based Milk

4. Sum portions across animal-based categories: Total animal-based portions/week = Red Meat + Poultry + Fish/Seafood + Eggs + Dairy

5. Calculate consumption frequency ratio: Animal-to-Plant Ratio = Total animal-based portions/week ÷ Total plant-based portions/week. This ratio serves as a descriptive indicator of relative consumption patterns within the sample, not as a validated nutritional comparison.

Worked Example: Low Plant-Based Protein Consumption Frequency

Food Category	Frequency Response	Portions/Week
Cooked Legumes	Once per week	1.0
Nuts and Seeds	2-4 times/week	3.0
Plant-Based Meat	Rarely or never	0
Plant-Based Milk	Rarely or never	0
**Total Plant-Based**		**4.0 portions/week**
Red Meat	Once per week	1.0
Poultry	2-4 times/week	3.0
Fish/Seafood	Once per week	1.0
Eggs	Once per day	7.0
Dairy	2-4 times/week	3.0
**Total Animal-Based**		**15.0 portions/week**
**Animal-to-Plant Ratio**		**15.0 ÷ 4.0 = 3.75:1**

Classification: low plant-based protein consumption frequency (4 portions/week falls within the 0-7 portions/week which would be classified as low frequency in our tertile-based categorization).

Notes:

1. The ratio of 3.75:1 indicates that this participant consumes animal-based protein sources approximately 3.75 times more frequently than plant-based sources within our frequency measurement framework. This ratio is a descriptive pattern indicator and should not be interpreted as a quantitative nutritional comparison.
2. This FFQ measures consumption frequency patterns of selected food categories, not validated protein intake in grams or nutritional adequacy. The primary outcomes are total portions per week for plant-based and animal-based protein sources and the ratio between them. All analyses in the main manuscript use these frequency values to examine relative consumption patterns within the sample, not to estimate absolute protein intake or assess nutritional requirements.

5. Data Quality Procedures

Step 1: Completeness Check.

- All verified FFQ items are completed for each participant.
- No missing data is allowed for consumption frequency calculation.
- Result: All 398 participants had complete FFQ data.

Step 2: Logical Consistency Checks.

- If participant reported "Never" for all plant sources → Flag for review.
- If total consumption frequency <3 portions/week or >70 portions/week → Flag for review.
- If participant in "Action/Maintenance" stage but plant consumption frequency <8 portions/week → Flag for review.
- Flags generated: 12 participants (3.0%).
- All flags reviewed; patterns deemed plausible.

Step 3: Outlier Detection and Capping.

Applied cap at 35 portions/week per food category during data collection.

Results:

- Total weekly consumption frequency ranged from 3 to 70 portions/week.
- Mean ± SD: 42.0 ± 16.8 portions/week.
- Distribution showed moderate positive skew (skewness = +0.67).
- No values exceeded plausible frequency limits after capping was applied.

6. Key Limitations

1. Incomplete Food Coverage

The adapted FFQ focused on discrete, identifiable plant protein sources. Grains, vegetables, and mixed dishes were not systematically captured, which likely resulted in underestimation of total plant-based protein consumption **frequency**.

Impact: Substantial underestimation of actual consumption frequency of plant-based protein sources (estimated 30-50% below true values). However, relative rankings across participants likely preserved.

2. Frequency-Based Measurement vs. Validated Nutritional Assessment

This approach measures consumption frequency patterns of selected food categories rather than validated protein intake in grams or nutritional adequacy. The FFQ was designed to capture relative differences in consumption frequency patterns across participants, not to provide precise quantitative estimates of protein intake or assess whether participants meet nutritional requirements.

Impact: Findings reflect relative consumption frequency patterns within the sample and cannot be used to draw conclusions about absolute protein intake levels or nutritional adequacy.

3. Portion Size Assumptions.

Standard portions were not validated through direct observation or photographic aids in the Saudi population. Actual consumed portions may differ from standard assumptions.

Impact: While portion size variation affects the hypothetical protein content that could be calculated from frequency data, our analysis uses only the frequency data itself (portions/week), making this limitation less critical for the frequency-based comparisons presented in the study.

4. Self-Report Bias.

Social desirability bias, recall bias, and lack of biomarker validation are inherent limitations of FFQ methodology.

5. Lack of Formal Validation.

Although the adapted FFQ underwent cultural adaptation through expert review, forward-backward translation, and pilot testing, it was not formally validated against biomarkers or comprehensive dietary records in this population. **Additionally, the FFQ was not validated specifically for measuring consumption frequency patterns in the Saudi Gen Z population.**

7. References

1. Hagmann D, Siegrist M, Hartmann C. Meat avoidance: motives, alternative proteins and diet quality in a sample of Swiss consumers. Public Health Nutr. 2019;22(13):2448-59. https://doi.org/10.1017/S1368980019001277

2. Subar AF, Freedman LS, Tooze JA, Kirkpatrick SI, Boushey C, Neuhouser ML, ... Krebs-Smith SM. Addressing current criticism regarding the value of self-report dietary data. J Nutr. 2015;145(12):2639-2645. https://doi.org/10.3945/jn.115.219634

3. Willett WC. Nutritional Epidemiology. 3rd ed. New York: Oxford University Press; 2013.

4. Thompson FE, Subar AF. Dietary assessment methodology. In: Coulston AM, Boushey CJ, Ferruzzi MG, eds. Nutrition in the Prevention and Treatment of Disease. 3rd ed. Academic Press; 2013:5-46.

5. Cade J, Thompson R, Burley V, Warm D. Development, validation and utilisation of food-frequency questionnaires—a review. Public Health Nutr. 2002;5(4):567-87. https://doi.org/10.1079/PHN2001318

6. Hu FB, Stampfer MJ, Rimm E, et al. Dietary fat and coronary heart disease: a comparison of approaches for adjusting for total energy intake and modeling repeated dietary measurements. Am J Epidemiol. 1999;149(6):531-40. https://doi.org/10.1093/oxfordjournals.aje.a009849
