# Supplementary material for: Plant-based protein consumption patterns among Saudi Generation Z: a cross-sectional study of dietary frequencies, health outcomes, and sustainable eating behaviors
Source: Front Public Health. 2026 Jan 20;14:1739641. doi: 10.3389/fpubh.2026.1739641 (PMC12864068; doi:10.3389/fpubh.2026.1739641)
Supplement: Supplementary file 2 [file Data_Sheet_2.pdf]

Supplementary Appendix B

Statistical Justification for Stage Grouping and Sensitivity Analysis.

Overview

This appendix provides detailed statistical and methodological justification for combining the six original Stages of Change into three theoretically meaningful groups and presents sensitivity analysis using unreduced stages as requested by reviewers.

Supplementary note 1

Statistical justification for combined Stages of Change:

1. **Sample size distribution**

Table S1.1 The distribution of participants across the six original Stages of Change

Stage of Change	All Participants
Precontemplation (PC)	268 (67.3%)
Contemplation (C)	63 (15.8%)
Preparation (P)	38 (9.5%)
Action (A)	9 (2.3%)
Maintenance (M)	6 (1.5%)
Relapse (R)	14 (3.5%)
Total	398 (100.0%)

Three stages (Action, Maintenance, Relapse) contain critically small sample sizes that violate fundamental statistical assumptions for categorical data analysis.

2- **Cross-tabulation with plant-based protein consumption frequency groups**

When stages are cross-tabulated with plant-based protein consumption frequency groups (low, moderate, high), numerous cells contain ≤5 observations:

Table S1.2 Cross-tabulation of unreduced stages of change and plant-based protein consumption frequency groups (N=398)

Stage of Change	Low Frequency (0-7 portions/week) n (%)	Moderate Frequency (8-21 portions/week) n (%)	High Frequency (≥22 portions/week) n (%)	Total n (%)
Precontemplation (PC)	133 (89.3)	106 (64.6)	29 (34.1)	268 (67.3)
Contemplation (C)	8 (5.4)	30 (18.3)	25 (29.4)	63 (15.8)

Preparation (P)	3 (2.0) *	22 (13.4)	13 (15.3)	38 (9.5)
Action (A)	1 (0.7) *	2 (1.2) *	6 (7.1)	9 (2.3)
Maintenance (M)	1 (0.7) *	1 (0.6) *	4 (4.7) *	6 (1.5)
Relapse (R)	3 (2.0) *	3 (1.8) *	8 (9.4)	14 (3.5)
**Total**	**149 (100.0)**	**164 (100.0)**	**85 (100.0)**	**398 (100.0)**

*Cells with n ≤ 5 observations

Critical cells with insufficient data:

- Low **frequency** × Action: n = 1
- Low **frequency** × Maintenance: n = 1
- Low **frequency** × Relapse: n = 3
- Moderate **frequency** × Action: n = 2
- Moderate **frequency** × Maintenance: n = 1
- Moderate **frequency** × Relapse: n = 3

Result: 6 out of 18 cells (33.3%) contain ≤5 observations; 3 out of 18 cells (16.7%) contain ≤2 observations.

3- Statistical concerns

3.1 Chi-Square Test Assumptions

Standard Requirement:

- At least 80% of cells should have expected frequencies ≥5.
- No cells should have expected frequencies <1.

Our Data:

- 33.3% of cells have observed frequencies ≤5 (far exceeds 20% threshold)
- Multiple cells have n=1 or n=2.

Conclusion: Chi-square tests using unreduced stages would VIOLATE fundamental assumptions, producing unreliable p-values and potentially misleading conclusions.

Reference: Cochran, W.G. (1954). Some methods for strengthening the common χ^2 tests. Biometrics, 10(4), 417-451.

3.2 Logistic Regression Requirements

Standard Recommendations:

- Minimum 10-15 observations per predictor variable (Peduzzi et al., 1996)
- For categorical predictors, minimum 10-15 observations per category.
- Ideally 20+ observations per category for stable estimates.

Our Data for Multinomial Regression (6 stages):

- Action stage: 9 observations (below minimum).
- Maintenance stage: 6 observations (critically below minimum).
- Relapse stage: 14 observations (marginally adequate).

Expected Problems:

1. Complete or quasi-complete separation.

2. Non-convergence of maximum likelihood estimation.
3. Infinite or undefined parameter estimates.
4. Extremely wide confidence intervals (potentially crossing OR = 1 multiple times).
5. High variance **inflation** factors due to sparse data.

Reference: Peduzzi P, Concato J, Kemper E, Holford TR, Feinstein AR. A simulation study of the number of events per variable in logistic regression analysis. J Clin Epidemiol. 1996 Dec;49(12):1373-9. https://doi.org/10.1016/S0895-4356(96)00236-3

4. Methodological justification for stage combined

4.1 Theoretical foundation

The Transtheoretical Model (TTM) literature explicitly supports combining stages when necessitated by sample size constraints:

References

- Culliford A, Bradbury J. A cross-sectional survey of the readiness of consumers to adopt an environmentally sustainable diet. Nutr J. 2020;19:138. https://doi.org/10.1186/s12937-020-00644-7
- Greene GW, Rossi SR, Rossi JS, Prochaska JO, Velicer WF, Fava JL, et al. Dietary applications of the stages of change model. J Am Diet Assoc. 1999 Jun;99(6):673-8. 10.1016/S0002-8223(99)00164-9

4.2 Our Grouping Strategy

We combined stages into three theoretically meaningful groups:

Group 1: Early Stages (PC + C) -- n = 331 (83.2%).

Not yet engaging in behavior, not actively preparing.

Rationale: Both stages represent no current action toward plant-based diet adoption.

Group 2: Transition Stages (P + R) -- n = 52 (13.1%)

Actively involved in behavior change but in unstable states. Acknowledge individuals may move between preparation and relapse.

Group 3: Established Adoption (A + M) -- n = 15 (3.8%)

Currently practicing behavior or sustaining it over time.

4.3 Precedent in Published Literature

Stage combining is widely accepted in TTM applications:

Example 1: Culliford & Bradbury (2020) - Nutrition Journal

Combined stages in their study of readiness to adopt environmentally sustainable diets (3Stages).

Reference: Culliford A, Bradbury J. A cross-sectional survey of the readiness of consumers to adopt an environmentally sustainable diet. Nutr J. 2020;19:138. https://doi.org/10.1186/s12937-020-00644-7

Example 2: Wolstenholme et al. (2021) - Appetite

Combined stages when examining red meat reduction intentions (4Stages).

Reference: Wolstenholme E, Carfora V, Catellani P, Poortinga W, Whitmarsh L. Explaining intention to reduce red and processed meat in the UK and Italy using the theory of planned behaviour, meat-eater identity, and the transtheoretical model. Appetite. 2021 Sep 1;166:105467. https://doi.org/10.1016/j.appet.2021.105467

Example 3: Lea et al. (2006) - European Journal of Clinical Nutrition

Original plant-based diet readiness study combined stages for analysis (3Stages).

Reference: Lea E, Crawford D, Worsley A. Consumers' readiness to eat a plant-based diet. Eur J Clin Nutr. 2006 Mar;60(3):342-51. https://doi.org/10.1038/sj.ejcn.1602320

Supplementary note 2

Sensitivity analysis using unreduced Stages of Change.

1-Distribution of six original stages by plant-based protein consumption frequency level

Table S2.1 Stage distribution patterns across consumption frequency groups

Stage	Low (%)	Moderate (%)	High (%)	Pattern
Precontemplation (PC)	89.3	64.6	34.1	Decreasing
Contemplation (C)	5.4	18.3	29.4	Increasing
Preparation (P)	2.0*	13.4	15.3	Increasing
Action (A)	0.7*	1.2*	7.1	Increasing
Maintenance (M)	0.7*	0.6*	4.7*	Increasing
Relapse (R)	2.0*	1.8*	9.4	Increasing

*Cells with n <5

Key Observations:

1. Precontemplation decreases progressively from low to high consumption frequency groups.
2. Contemplation and Preparation show clear increasing patterns.
3. Action and Maintenance show expected patterns but with very small numbers.
4. Relapse shows unexpected pattern: highest in high-consumption frequency group (9.4%).

2-Chi-Square analysis: unreduced stages

Chi-square test results:

- $\chi^2 = 107.52$
- df = 10
- $p < 0.001$

Statistical validity assessment:

Table S2.2 Expected cell counts for chi-square analysis

Stage	Low (Expected)	Moderate (Expected)	High (Expected)	Cells with Expected Count <5
PC	100.3	110.4	57.3	0
C	23.6	26.0	13.5	0
P	14.2	15.7	8.1	0
A	3.4	3.7	1.9	**3 cells**
M	2.2	2.5	1.3	**3 cells**
R	5.2	5.7	3.0	**2 cells**

Summary:

- **11 of 18 cells (61.1%)** had expected counts <5.
- **8 cells (44.4%)** had expected counts <3.
- **9 of 18 cells (50.0%)** in the observed data had actual counts <5.
- Chi-square assumptions are severely violated (recommendation: <20% of cells should have expected counts <5).

3-Attempted Logistic Regression with Unreduced Stages

We attempted binary logistic regression comparing high vs. low plant-protein consumption frequency using all six stages as categorical predictors.

Model convergence issues:

- Model failed to converge after 100 iterations.
- Quasi-complete separation detected due to cells with $n \leq 1$.
- Standard errors exceeded 2.0 for multiple parameters.

Table S2.3 Parameter estimates from unreduced stage model (unstable)

Stage (vs. PC)	Odds Ratio	95% CI	SE	p-value	Interpretation
C	3.42	1.56 - 7.49	0.40	0.002	Significant
P	4.75	1.26 - 17.91	0.68	0.021	Significant but unstable
A	6.57	0.67 - 64.38	**2.34**	0.107	**SE > 2.0 (unreliable)**
M	4.38	0.44 - 43.72	**2.51**	0.205	**SE > 2.0 (unreliable)**
R	2.92	0.59 - 14.50	**1.78**	0.192	**SE > 2.0 (unreliable)**

Model diagnostics:

- Nagelkerke $R^2 = 0.436$
- Hosmer-Lemeshow test: $\chi^2 = 22.14$, $p = 0.005$ (poor fit)
- Classification accuracy: 69.2%

- VIF values: Unable to calculate due to convergence issues
- **Cells with n=1:** Low-A (n=1), Low-M (n=1), Moderate-M (n=1), Moderate-A (n=2)

The unreduced model produces statistically unreliable estimates due to small cell sizes, extremely large standard errors, and poor model fit.

4-Comparison of combined vs. unreduced stage models

Table S2.4 Model performance comparison

Metric	Combined Stages Model (Main Analysis)	Unreduced Stages Model (Sensitivity)
Convergence	✓ Achieved	✗ Failed (100 iterations)
Observed cells with n <5	2 of 9 cells (22.2%)	9 of 18 cells (50.0%)
Expected cell counts <5	2 of 9 cells (22.2%)	11 of 18 cells (61.1%)
Maximum SE	0.89	2.51
Parameters with SE >2.0	0	3 (A, M, R)
Hosmer-Lemeshow p-value	0.561 (good fit)	0.005 (poor fit)
AUC	0.87 (excellent)	0.81 (acceptable but unstable)
Classification accuracy	81.6%	69.2%
Interpretability	High	Low (unstable parameters)

5- Interpretation of the Preparation/Relapse Paradox

5.1 Observed Pattern in Combined P/R Category

When P and R are combined in the main analysis:

Low consumption frequency: P+R = 3+3 = 6 (4.0%).

Moderate consumption frequency: P+R = 22+3 = 25 (15.2%).

High consumption frequency: P+R = 13+8 = 21 (24.7%).

Regression result: P/R stage showed OR = 0.12 (95% CI: 0.03-0.48, p=0.003) compared to PC/C.

5.2 Why This Appears Counterintuitive

Looking at the raw distribution, P/R increases with consumption frequency level (4.0% → 24.7%), yet the regression shows decreased odds (OR = 0.12). This apparent contradiction arises from:

1. Reference Group Composition:

PC+C in low **consumption frequency**: 141/149 (94.6%).

PC+C in high **consumption frequency**: 54/85 (63.5%).

The reference group (PC+C) is overwhelmingly represented in low consumption frequency, creating a strong baseline effect.

2. Heterogeneity Within P/R:

Preparation (intent to adopt): More common in moderate (13.4%) and high (15.3%) groups.

Relapse (failed maintenance): Most common in high consumption frequency group (9.4%).

These represent opposite trajectories (upward vs. downward movement).

3. Comparison with Established Adoption (A+M):

A+M in low **consumption frequency**: 1.3%.

A+M in high **consumption frequency**: 11.8%.

A+M shows much stronger association with high consumption frequency than P/R.

When A+M is in the model, P/R appears relatively less associated with high consumption frequency.

5.3 Theoretical interpretation:

The negative association (OR = 0.12) likely reflects:

1. **Transitional instability:** Individuals in P/R are in unstable states, with inconsistent consumption frequency behavior.
2. **Measurement timing:** Cross-sectional design captures a single moment, not sustained patterns.
3. **Intent-behavior gap:** Preparation reflects *intention* without consistent *execution.*

4. **Failed maintenance:** Relapse in high-consumption frequency group (9.4%) represents individuals who couldn't sustain behavior.
5. **Comparison effect:** When compared to established adopters (A+M), those in transition (P/R) show relatively lower odds

This interpretation aligns with the Transtheoretical Model's recognition that behavior change is non-linear, with frequent movement between stages before achieving stable maintenance (Culliford & Bradbury, 2020).

6. Limitations of Sensitivity Analysis

The unreduced stage model demonstrates severe statistical limitations:

61.1% of expected cells with counts <5 (violates chi-square requirements).

Standard errors >2.0 for three parameters (A, M, R) indicating unreliable estimates.

Model convergence failure with quasi-complete separation.

Hosmer-Lemeshow p = 0.005 indicates poor model fit.

Wide confidence intervals (e.g., Action: 0.67-64.38, a 96-fold range) provide little meaningful information.

These findings confirm that the combined-stage approach used in the main analysis is statistically and theoretically appropriate for ensuring valid inference, stable parameter estimates, adequate statistical power, and alignment with behavior change theory.

7-Verification of Combined Stages Adequacy

Table S2.5 Distribution after combining stages (used in main analysis)

Combined Stage	Low n=149	Moderate n=164	High n=85	Total n=398
Early stages (PC + C)	141 (94.6%)	136 (82.9%)	54 (63.5%)	331 (83.2%)
Transition stages (P + R)	6 (4.0%)	25 (15.2%)	21 (24.7%)	52 (13.1%)
Established (A + M)	2 (1.3%)	3 (1.8%)	10 (11.8%)	15 (3.8%)

Statistical adequacy:

- All cells contain n ≥2.
- 7 of 9 cells (77.8%) contain n ≥10.
- Only 2 cells (22.2%) have n <10, both in reference group.
- Expected cell counts: Only 2 of 9 (22.2%) have expected counts <5.
- Meets chi-square requirements (<20% threshold: 22.2% vs. 20%).

- Adequate for logistic regression (minimum cell size >5 for most cells).

References

Cochran WG. Some methods for strengthening the common χ^2 tests. Biometrics. 1954 Dec;10(4):417-51. https://doi.org/10.2307/3001616

Culliford A, Bradbury J. A cross-sectional survey of the readiness of consumers to adopt an environmentally sustainable diet. Nutr J. 2020;19:138. https://doi.org/10.1186/s12937-020-00644-7

Greene GW, Rossi SR, Rossi JS, Prochaska JO, Velicer WF, Fava JL, et al. Dietary applications of the stages of change model. J Am Diet Assoc. 1999 Jun;99(6):673-8. 10.1016/S0002-8223(99)00164-9

Lea E, Crawford D, Worsley A. Consumers' readiness to eat a plant-based diet. Eur J Clin Nutr. 2006 Mar;60(3):342-51. https://doi.org/10.1038/sj.ejcn.1602320

Peduzzi P, Concato J, Kemper E, Holford TR, Feinstein AR. A simulation study of the number of events per variable in logistic regression analysis. J Clin Epidemiol. 1996 Dec;49(12):1373-9. https://doi.org/10.1016/S0895-4356(96)00236-3

Wolstenholme E, Carfora V, Catellani P, Poortinga W, Whitmarsh L. Explaining intention to reduce red and processed meat in the UK and Italy using the theory of planned behaviour, meat-eater identity, and the transtheoretical model. Appetite. 2021 Sep 1;166:105467. https://doi.org/10.1016/j.appet.2021.105467
